# GCN2 and TOR converge on aging

**DOI:** 10.18632/aging.100586

**Published:** 2013-08-05

**Authors:** Anna Vlanti, Aris Rousakis, Popi Syntichaki

**Affiliations:** ^1^ Biomedical Research Foundation of the Academy of Athens, Center of Basic Research II, Athens, Greece; ^2^ School of Medicine, University of Athens, Athens, Greece

Coordinated responses in all tissues upon nutrient stress ensure the survival of an organism in periods of food unavailability. Two evolutionarily conserved, nutrient-sensing signaling pathways that promote stress adaptation following starvation are the general amino acid control pathway that activates GCN2 kinase and the target of rapamycin (TOR) kinase pathway [1]. In response to amino acid deprivation GCN2 is activated, upon binding of uncharged tRNAs, whereas TOR is inhibited through regulation of its localization. In both cases the outcome is reduction of global protein synthesis, albeit different mechanisms are involved. GCN2 directly phosphorylates the eukaryotic initiation factor 2α (eIF2α), whereas inactivation of TOR dephosphorylates the ribosomal protein S6 kinase (S6K) and the eukaryotic translation initiation factor 4E (eIF4)-binding protein (4E-BP). The result is repression of translation initiation of most mRNAs, accompanied by favored translation of specific mRNAs.

Translational control of gene expression in response to nutrient and other environmental stresses contributes to stress management via energy saving and selective synthesis of stress-responsive proteins [2]. These effects might be also responsible for lifespan extension through down-regulation of mRNA translation. Inhibition of TOR signaling, by genetic or pharmacological means, has been associated with treatment of several diseases and increased lifespan in many organisms, including humans [3]. Similarly, a robust nutritional intervention that slows aging and its related pathology in diverse species is dietary restriction (DR), the effects of which, at the molecular level, are attributed largely to TOR inhibition. In consistence, deficiency of downstream targets of TOR such as S6K or translation factors/regulators extends lifespan in model systems [4]. Also, under conditions of impaired TOR, the activation of autophagy, a catabolic process that enhances degradation and recycling of damaged cellular components during ageing, contributes to longevity. Interestingly, regulation of autophagy in response to nutrient starvation involves GCN2 signaling, in yeast and mammalian cells [5].

**Figure 1:**
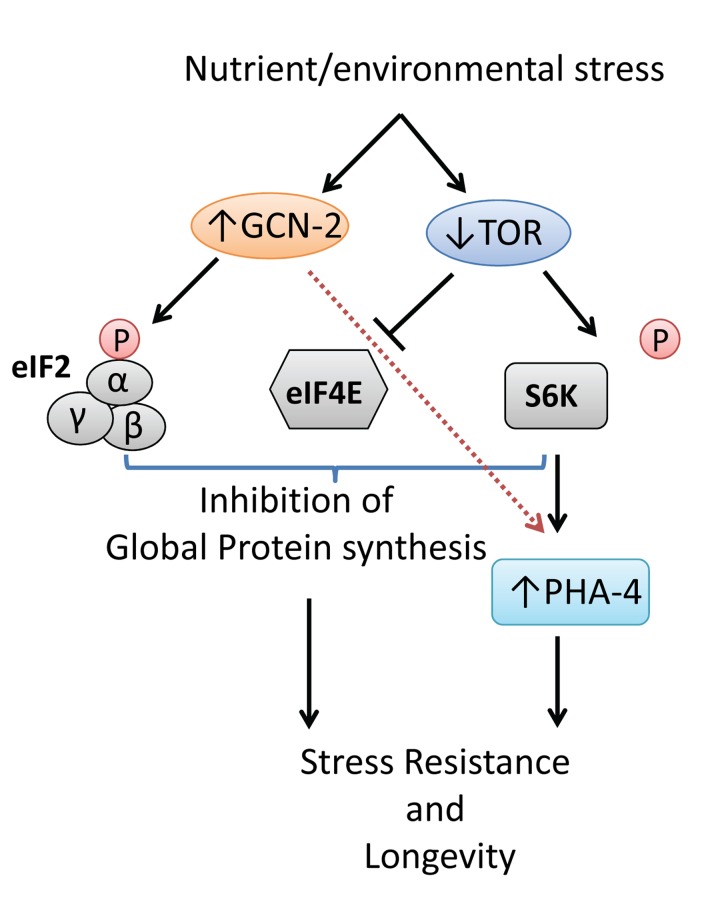
Model depicting the function of GCN-2 in response to nutrient or environmental stress and its impact on survival and longevity of *C. elegans*.

Despite that both GCN2 and TOR can sense nutrient deprivation and regulate protein synthesis and autophagy, the impact of GCN2 signaling in longevity and its connection to TOR is not clear. Evidence in mice indicates that reduced amino acid levels activate GCN-2 and can suppress TOR activity on its targets S6K and 4E-BP. In our recent work [6], we showed that GCN-2 in *Caenorhabditis elegans* can influence the lifespan of nutrient-sensitized worms, through regulation of TOR signaling. Deletion of *gcn-2* had no effect on growth and lifespan of wild-type animals under normal conditions but reduced their lifespan under amino acid limitation, as this was recapitulated through RNAi-mediated silencing of aminoacyl-tRNA synthetases genes. Moreover, disruption of *gcn-2* decreased the long-life of *eat-2* mutants (a genetic model of DR) and of TOR-defective worms. Longevity conferred by inactivation of the downstream target of TOR *rsks-1*/S6K, but not of *ife-2*/eIF4E, was also affected by GCN-2 loss. In *C. elegans*, the TOR-S6K signaling antagonizes a forkhead transcription factor, PHA-4/FoxA, with roles in early development, survival of first stage (L1) larvae under starvation and DR-mediated longevity of adults [7, 8]. We revealed that GCN-2 was required for the induction of *pha-4* under conditions of amino acid deprivation or TOR inactivation. Consequently, the expression of known PHA-4 target genes, involved in stress defense and autophagy, was induced in a GCN-2-dependent manner thus contributing to stress survival and longevity of worms under nutrient or other stress (Figure 1).

Although the ways in which PHA-4 regulates lifespan are not well-defined they may be relevant to the mechanisms used by L1 larvae to survive starvation [8]. PHA-4 has a broad role in regulation of gene expression but can preferentially bind to specific set of genes under distinct developmental or environmental conditions. Among the candidate PHA-4 targets in starved larvae are stress-responsive and autophagic genes, some of which are PHA-4-regulated in dietary restricted adults, suggesting common mechanisms. Intriguingly, genes involved in metabolic processes such as many nuclear hormone receptors (NHRs) and multiple regulators of sterol and fatty-acid metabolism constitute candidate targets of PHA-4 in starved larvae. Lipid metabolism and fat storage occurs mostly in the intestine of worms, where PHA-4 is induced upon DR, and has been linked to lifespan regulation. Thus, alterations in metabolic pathways in specific tissues, beyond cellular protection, could contribute to longevity induced by TOR inactivation during nutrient stress, but their dependence on GCN-2 remains to be addressed. Also, important questions remain with respect to the interplay between the GCN-2 and other stress signaling pathways that modulate lifespan under such conditions. Elucidation of the above is of great interest, as certain amino acid-restricted diets in rodents extend lifespan, improve their metabolic traits and confer protection from various age-related diseases.
